# The Seed Repair Response during Germination: Disclosing Correlations between DNA Repair, Antioxidant Response, and Chromatin Remodeling in *Medicago truncatula*

**DOI:** 10.3389/fpls.2017.01972

**Published:** 2017-11-14

**Authors:** Andrea Pagano, Susana de Sousa Araújo, Anca Macovei, Paola Leonetti, Alma Balestrazzi

**Affiliations:** ^1^Department of Biology and Biotechnology ‘Lazzaro Spallanzani’, University of Pavia, Pavia, Italy; ^2^Plant Cell Biotechnology, Instituto de Tecnologia Química e Biológica António Xavier (ITQB-NOVA), Oeiras, Portugal; ^3^Institute for Sustainable Plant Protection, National Research Council (CNR), Bari, Italy

**Keywords:** antioxidant response, chromatin remodeling, comet assay, DNA repair, *Medicago truncatula*, seed germination, trichostatin A

## Abstract

This work provides novel insights into the effects caused by the histone deacetylase inhibitor trichostatin A (TSA) during *Medicago truncatula* seed germination, with emphasis on the seed repair response. Seeds treated with H_2_O and TSA (10 and 20 μM) were collected during imbibition (8 h) and at the radicle protrusion phase. Biometric data showed delayed germination and impaired seedling growth in TSA-treated samples. Comet assay, performed on radicles at the protrusion phase and 4-days old *M. truncatula* seedlings, revealed accumulation of DNA strand breaks upon exposure to TSA. Activation of DNA repair toward TSA-mediated genotoxic damage was evidenced by the up-regulation of *MtOGG1*(*8-OXOGUANINE GLYCOSYLASE/LYASE*) gene involved in the removal of oxidative DNA lesions, *MtLIGIV*(*LIGASE IV*) gene, a key determinant of seed quality, required for the rejoining of DNA double strand breaks and *TDP(TYROSYL-DNA PHOSPHODIESTERASE)* genes encoding the multipurpose DNA repair enzymes tyrosyl-DNA phosphodiesterases. Since radical scavenging can prevent DNA damage, the specific antioxidant activity (SAA) was measured by DPPH (1,1-diphenyl-2-picrylhydrazyl) and Folin-Ciocalteu reagent assays. Fluctuations of SAA were observed in TSA-treated seeds/seedlings concomitant with the up-regulation of antioxidant genes *MtSOD(SUPEROXIDE DISMUTASE, MtAPX*(*ASCORBATE PEROXIDASE*) and *MtMT2*(*TYPE 2 METALLOTHIONEIN*). Chromatin remodeling, required to facilitate the access of DNA repair enzymes at the damaged sites, is also part of the multifaceted seed repair response. To address this aspect, still poorly explored in plants, the *MtTRRAP*(*TRANSFORMATION/TRANSACTIVATION DOMAIN-ASSOCIATED PROTEIN*) gene was analyzed. TRRAP is a transcriptional adaptor, so far characterized only in human cells where it is needed for the recruitment of histone acetyltransferase complexes to chromatin during DNA repair. The *MtTRRAP* gene and the predicted interacting partners *MtHAM2* (*HISTONE ACETYLTRANSFERASE OF THE MYST FAMILY*) and *MtADA2A* (*TRANSCRIPTIONAL ADAPTOR*) showed tissue- and dose-dependent fluctuations in transcript levels. PCA (Principal Component Analysis) and correlation analyses suggest for a new putative link between DNA repair and chromatin remodeling that involves *MtOGG1* and *MtTRRAP* genes, in the context of seed germination. Interesting correlations also connect DNA repair and chromatin remodeling with antioxidant players and proliferation markers.

## Introduction

Fast and uniform seed germination and successful seedling establishment represent nowadays a priority for gaining high crop yields. Within this context, the availability of molecular hallmarks of seed vigor is expected to positively impact seed technology, providing innovative tools to overcome the pitfalls of conventional priming protocols (Paparella et al., 2015; Araújo et al., 2016a; Macovei et al., 2016). The seed repair response includes the early activation of antioxidant mechanisms and DNA repair pathways that significantly contribute to define the final seed/seedling value in terms of germination rate and robustness. Indeed, seed imbibition triggers the pre-germinative metabolism characterized by intense DNA repair, essential premise to *de novo* DNA synthesis in embryo cells (Ashraf and Bray, 1993). Up-regulation of DNA repair genes during early seed imbibition has been documented in *Arabidopsis* (Waterworth et al., 2009, 2010) and in *Medicago truncatula* (Macovei et al., 2010, 2011; Balestrazzi et al., 2011) while the crucial role of ATM (Ataxia Telangiectasia Mutated) kinase in maintaing genome stability in seeds has been recently demonstrated (Waterworth et al., 2016). It is also known that major transcriptional changes and chromatin rearrangements mark the developmental transition during seed germination (Tanaka et al., 2008; Boychev et al., 2014; Wang et al., 2016). Key players in chromatin remodeling are histone deacetylases (HDACs) that remove acetyl groups from histones, facilitating chromatin condensation and consequently gene silencing (Grandperret et al., 2014) while histone acetyltransferases (HATs) carry out the transfer of acetyl groups to the lysine residues at the N-terminal region of histones and interact with transcription factors, triggering gene expression (Boychev et al., 2014). The involvement of specific HDACs in the molecular networks underlying seed germination and early seedling development has been reported as in the case of HDA19/HD1 which participates in the transcriptional repression of the *AtABI3 (ABSCISIC ACID INSENSITIVE)* gene promoter during early seedling development in *Arabidopsis*. As a consequence, the ABA (abscisic acid) signaling pathway is suppressed, allowing the establishment of young seedlings (Ryu et al., 2014).

Chromatin remodeling is one of the highly conserved pathways that contribute to efficient DNA repair in eukaryotes (Gursoy-Yuzugullu et al., 2016). Chromatin remodelers disrupt DNA-histone interactions, allowing the access of the DNA repair machinery at the damaged site and controlling the temporal and spatial steps of the process (Menoni et al., 2016). The role of chromatin remodeling in the DNA damage response (DDR) is currently an hot issue in plants (Donà and Mittelsten Scheid, 2015). In a recent work performed on *Arabidopsis* cell suspension cultures, Gonzalez-Arzola et al. (2017) reported that the *Arabidopsis* histone chaperone NIRP1 (NAP(NUCLEOSOME ASSEMBLY PROTEIN)-RELATED PROTEIN) binds chromatin following DNA breaks accumulation, facilitating nucleosome disassembling and the interaction of repair enzymes with DNA lesions. Despite the expanding knowledge on the interplay between DDR and chromatin remodeling in plants, several aspects remain still uncovered, such as the role played by the transcriptional activator TRRAP (TRANSFORMATION/TRANSACTIVATION DOMAIN-ASSOCIATED PROTEIN), found in the HAT complexes SAGA/TFTC (SPT-ADA-GCN5 acetyltransferase/TBP-free-TAF-complex) and TFTC/STAGA (SPT3-TAF9-GCN5 acetyltransferase) of *Drosophila melanogaster* and human cells (Brown et al., 2000). TRRAP allows the recruitment of HAT complexes to chromatin during transcription, replication, and DNA repair (Murr et al., 2007), with a peculiar role in double strand breaks (DSBs) repair. It has been hypothesized that DDR components might preferentially recruit the TRRAP-containing HAT complexes at the DSBs sites. It is also possible that DSBs-induced DDR networks result in chromatin alterations, such as the presentation of methylated lysine 79 of histone H3, thus facilitating the binding of TRRAP-containing HAT complexes at the damaged site (Huyen et al., 2004).

HDAC inhibitors, such as trichostatin A (TSA), induce ROS (reactive oxygen species) accumulation and cause DNA damage, providing the opportunity of investigating the biological significance of chromatin rearrangements in a genotoxic stress context (Robert et al., 2016). The molecular events that characterize early seed germination represent an intriguing model for exploring the link between chromatin remodeling and DNA repair in plants. In the present work, we show novel insights into the effects caused by TSA in germinating seeds of the model legume *M. truncatula*, focusing on genotoxic injury and exploring the multifaceted seed repair response in terms of expression profiles of DDR, antioxidant, and chromatin remodeling genes.

## Materials and methods

### Plant materials and treatments

*M. truncatula* seeds (commercial genotype, kindly provided by Dr. Ana Barradas, Fertiprado L.d.a., Vaiamonte-Monforte, Portugal) were transferred to Petri dishes containing two filter papers moistened with 2.5 ml of dH_2_O (control, CTRL), sealed and kept in a growth chamber at 22°C under light conditions with photon flux density of 150 μmol m^−2^ s^−1^, photoperiod of 16/8 h and 70–80% relative humidity. Similarly, for treatments with TSA (TSA, Sigma-Aldrich, Milan, Italy), seeds were sown over filter paper imbibed with 2.5 ml of 10 μM and 20 μM TSA solutions, and hereby referred to as TSA10 and TSA20 samples. TSA-treated and untreated seeds were germinated in parallel, under the above mentioned conditions. Seeds were mantained moistered by addition of water whenever needed during the study. Seeds with protrusion of the primary radicle were considered germinated and counted 8-days after imbibition. The peak value was calculated as the highest mean daily germination (MDG) reached at any time during the germination test (Ranal and Garcia de Santana, 2006). Germination parameters were analyzed in three independent replicates (with 20 seeds each) for each treatment. *M. truncatula* seeds and seedlings were harvested at the indicated time points, the fresh weight was measured and samples were stored in liquid N_2_ for molecular analyses.

### Comet assay

Nuclei were extracted from *M. truncatula* tissues (radicles isolated at the protrusion phase and 4-days old seedlings, respectively) as described by Gichner et al. (2000). The suspension containing the purified nuclei and a solution containing 1% low melting point agarose (Sigma-Aldrich) in phosphate-buffered saline (PBS) at 37°C were mixed in equal volume. Two drops of the resulting suspension were then pipetted onto agarose pre-coated slides and solidified on ice. Slides were incubated for 20 min at r.t. in high salt lysis buffer (2.5 M NaCl, 100 mM Tris-HCl pH 7.5, 100 mM EDTA) to disrupt the nuclear membrane. For alkaline comet assay, nuclei were denatured in alkaline buffer (1 mM Na_2_EDTA, 300 mM NaOH, pH > 13) for 30 min at 4°C and then electrophoresed in the same buffer for 25 min at 0.72 V cm^−1^ in a cold chamber. After electrophoresis, slides were washed twice in 0.4 M Tris-HCl pH 7.5 for 5 min, rinsed once in 70% ethanol (v/v) for 12 min at 4°C and dried at r.t. overnight. Exposure to alkaline conditions causes DNA unwinding and visualization of single strand breaks. Subsequently, slides were stained with 20 μL DAPI (4′-6-Diamidine-2′-phenylindole dihydrochloride; 1 μg ml^−1^, Sigma-Aldrich). For each slide, one hundred nucleoids were scored, using a fluorescence microscope with an excitation filter of 340–380 nm and a barrier filter of 400 nm. Nucleoids were classified and results were expressed in arbitrary units (a.u.) according to Collins (2004).

### DPPH (1, 1-Diphenyl-2-Picrylhydrazyl) test and folin-ciocalteu reagent assay

Seed and seedling extracts were prepared as described by Li et al. (2008). Treated and control samples (100 mg each) were homogenized to fine powder in 1 ml 80% acetone. Samples were incubated overnight at 23°C under gentle shaking, then stored at −20°C until use. The free radical-scavenging activity or antioxidant potential of extracts was determined by DPPH test which exploits the reactivity of the DPPH radical with antioxidant compounds (Braca et al., 2001). Aliquots (0.1 ml each) of diluted extract (1:5 and 1:10 in 80% acetone) were added to 3 ml of a solution containing 100 mM DPPH (Sigma-Aldrich) dissolved in methanol. The reaction was carried out in the dark at r.t. for 30 min. A standard curve was built, using 0.1 ml ascorbic acid (Sigma-Aldrich; concentrations in the 0.125–2.000 mM range). Three biological replicates consisting of a pool of seeds/seedlings (approximately of 100 mg) were used in this study. Two technical replicates were made. DPPH reduction was measured by the decrease in absorbance at λ = 517 nm, using a V-530 spectrophotometer (Jasco Inc. Mary's Ct, Easton, MD, U.S.A.). DPPH radical scavenging activity was calculated from the absorption according to the following equation: DPPH radical scavenging activity % = [(A_control_ – A_sample_/A_control_)] × 100. The antioxidant potential was expressed as ascorbic acid equivalents (AAE) ${\text{mg}}_{\text{dryweight}}^{-1}$. Total phenolic compounds were measured using the Folin-Ciocalteu reagent (Spanos and Wrolstad, 1990) and expressed as gallic acid equivalents (GAE) ${\text{mg}}_{\text{dryweight}}^{-1}$ by reference to a standard curve. Aliquots (20 μl each) of diluted extract (1:5 and 1:10 in 80% acetone) were mixed with distilled H_2_O (dH_2_O, 1.58 ml) and with the Folin-Ciocalteu reagent (0.1 ml; Sigma-Aldrich) under vigorous shaking. After incubation (8 min), the reaction was neutralized with 0.3 ml of 7.5% (w/v) Na_2_CO_3_ (Sigma-Aldrich) and samples were incubated for 120 min at r.t. in the dark. A calibration curve was built with gallic acid concentrations in the 50–500 mg l^−1^ range. Two technical replicates were used per reaction. The absorption of the resulting blue color was measured at λ = 765 nm, using a V-530 spectrophotometer (Jasco Inc.). The specific antioxidant activity (SAA) defined as the ratio between the antioxidant potential and the phenolic content was calculated and expressed as μg AAE mg^−1^ GAE.

### RNA extraction, cDNA synthesis and quantitative real-time polymerase chain reaction

RNA isolation was carried out as described by Oñate-Sanchez and Vicente-Carbajosa (2008). RNAs were extracted from three biological replicates consisting of a pool of seeds/seedlings (aproximately of 100 mg) each. cDNAs were obtained using the RevertAid First Strand cDNA Synthesis Kit (Thermofisher Scientific, Milan, Italy) according to the manufacturer's suggestions. Quantitative real-time polymerase chain reaction (*q*RT-PCR) was performed with the Maxima SYBR Green qPCR Master Mix (2X) (ThermoFisher Scientific) according to supplier's indications, using a Rotor-Gene 6000 PCR apparatus (Corbett Robotics Pty Ltd., Brisbane, Queensland Australia). Amplification conditions were as follows: denaturation at 95°C for 10 min, and 45 cycles of 95°C for 15 s and 60°C for 60 s. Oligonucleotide primers were designed using the Real-Time PCR Primer Design program from GenScript (https://www.genscript.com/ssl-bin/app/primer) and further validated through the online software Oligo Analyzer (https://eu.idtdna.com/calc/analyzer) (Table 1). The following genes were tested: *MtADA2A*(*TRANSCRIPTIONAL ADAPTOR*) (Medtr3g082790), *MtAPX*(*ASCORBATE PEROXIDASE*) (Medtr4g061140), *MtH4*(*HISTONE H4*) (Medtr4g128150), *MtHAM2*(*HISTONE ACETYLTRANSFERASE OF THE MYST FAMILY*) (Medtr3g007710), *MtHDA19/HD1*(*HISTONE DEACETYLASE*) (Medtr3g118535), *MtLIGIV*(*LIGASEIV*) (Medtr2g038030), *MtMT2*(*TYPE 2 METALLOTHIONEIN*) (Medtr8g060850), *MtOGG1*(*8-OXOGUANINE GLYCOSYLASE/LYASE*) (Medtr3g088510), *MtSOD*(*SUPEROXIDE DISMUTASE*) (Medtr7g114240), *MtTDP1*α (*TYROSYL-DNA PHOSPHODIESTERASE*) (Medtr7g050860), *MtTDP1*β (Medtr8g095490), *MtTDP2*α (Medtr4g132300), *MtTOP2*(*DNA TOPOISOMERASE 2*) (Medtr3g085840), *MtTOR*(*TARGET OF RAPAMYCIN*) (Medtr5g005380), and *MtTRRAP*(*TRANSFORMATION/TRANSACTIVATION DOMAIN-ASSOCIATED PROTEIN)* (Medtr5g022000.1). Sequences were retrieved using the NCBI gene database (https://www.ncbi.nlm.nih.gov/). For each oligonucleotide set, a no-template water control was used. Quantification was carried out using the *MtUBI*(*UBIQUITIN-LIKE*) (Medtr3g091400) and *MtPDF2*(*PROTODERMAL FACTOR 2*) (Medtr6g084690) as reference genes for the experimental conditions (treated vs. untreated) used in this work. The raw, background-subtracted fluorescence data provided by the Rotor-Gene 6000 Series Software 1.7 (Corbett Robotics) was used to estimate PCR efficiency (E) and threshold cycle number (C_t_) for each transcript quantification. The Pfaffl method (Pfaffl, 2001) was used for relative quantification of transcript accumulation and statistical analysis was performed with REST2009 Software V2.0.13 (Qiagen GmbH, Hilden, Germany). Heatmaps representing the Log 2 fold changes (Log2 FC) of mean transcript expression levels between TSA treatments were plotted using the data visualization tools available in the MultiExperiment Viewer (MeV) software (Howe et al., 2011).

**Table 1:** List of oligonucleotide primers used for *q*RT-PCR analyses.

**Gene**	**Forward Primer**	**Reverse Primer**	**Efficiency**
*MtADA2A*	5′-CCTCATAAAAGCAATCATCCGTATC-3′	5′-CAACATCATTCCAATTCCCAAATCC-3′	1.75
*MtAPX*	5′-AGCTCAGAGGTTTCATCGCT-3′	5′-CGAAAGGACCACCAGTCTTT-3′	1.76
*MtH4*	5′-TGCGCGATAACATCCAGGGAATC-3′	5′- ATACGCTTCACACCACCACGTC-3′	1.64
*MtHAM2*	5′-TGATGGCAAGAAGAACAAGG-3′	5′-AACCATGTGGCATCCTCTTT-3′	1.73
*MtHDA19/HD1*	5′-GCTCGGTGTTGGTGCTAT-3′	5′-TATTCATAATACTCGTGCTCTGGC-3′	1.72
*MtLIGIV*	5′-TCACAACCACACGAGACTGA-3′	5′-GCCCGATTCCCTTGTTTTGT-3′	1.75
*MtMT2*	5′-CATGTCAAGCTCATGCGGCAAC-3′	5′-TGCCGTAGTTGTTTCCCTTCCC-3′	1.72
*MtOGG1*	5′-AAACACCGCACCTTCTCAAT-3′	5′-TGTGGAGATGTTTGAGGGAA-3′	1.73
*MtSOD*	5′-CCTGAGGATGAGACTCGACA-3′	5′-GAACAACAACAGCCCTTCCT-3′	1.79
*MtTDP1*α	5′-ACGAGTTGGGAGTGCTCTTT-3′	5′-GGGATTTATCCTTCGATTGTTT-3′	1.63
*MtTDP1*β	5′-TGCCGGTTACAATTGCATGTCAG-3′	5′-AGTTTCAGGAAATGGAGGATGCAC-3′	1.72
*MtTDP2α*	5′-CAGATGTTCAGCAAGGAACG-3′	5′-CCCGTCTTGCAAAGGATATT-3′	1.74
*MtTOP2*	5′-AGGATCCGTGGGATTGTAAGGC-3′	5′-ACAACAGAGAGGCCAGCCATAG-3′	1.78
*MtTOR*	5′-TGATGTTACCGTACGCCACT-3′	5′-TAAAGCGGCAAATACTGCAC-3′	1.81
*MtTRRAP*	5′-GCGACTTTTGGCTGTGGTTA-3′	5′-AGAGACTGGGGAACTTCTGC-3′	1.73
*MtUBI*	5′-GCAGATAGACACGCTGGGA-3′	5′-AACTCTTGGGCAGGCAATAA-3′	1.81
*MtPDF2*	5′-GTGTTTTGCTTCCGCCGTT-3′	5′-CCAAATCTTGCTCCCTCATCTG-3′	1.78

*For each oligonucleotide set, PCR efficiency is reported*.

### Statistical analysis

For each phenological stage studied (dry seed, 8 h imbibition, radicle protrusion and 4-days old seedling) and variables (gene expression, biochemical parameters, comet assay) significant differences between TSA concentrations were determined with One-way ANOVA (Analysis of Variance) using the statistical software Statistica, version 6 (Statsoft). For each treatment, three biological replicates were considered. Means were then compared using the Tukey's HSD (Honest Significant Difference) test. Means with a significance value lower than 0.05 (*P*-value ≤ 0.05) were considered statistically different. Principal components analysis (PCA) was performed on molecular and biochemical variables quantified across the study. Variables included are content of total phenolic compounds (GAE), radical-scavenging activity (AAE), SAA, and the expression levels of genes involved in antioxidant response (*MtSOD, MtAPX* and *MtMT2*), proliferation and development (*MtH4, MtTOP2, MtHDA19/HD1*, and *MtTOR*), chromatin remodeling (*MtTRAPP, MtHAM2*, and *MtADA2A*) and DNA repair (*MtOGG1, MtLIGIV, MtTDP1*α, *MtTDP1*β, and *MtTDP2*α). Data were collected from CTRL, TSA10 and TSA20 samples that included dry seeds, imbibed seeds (8 h of imbibition), seeds collected at the radicle protrusion phase, and 4-days old seedlings. The standardized variables were subjected to PCA allowing the extraction of the rotated orthogonal components, as well as their relative scores and potential correlations among variables. Only principal components (PCs) with an eigenvalue >1 were considered for discussion. Additionally, regression analysis was conducted to extract significant correlations between studied variables. Correlations with a *P*-value ≤ 0.05 and *r* = Pearson correlation above 0.70 were considered for discussion. Multivariate and product-moment correlation analyses were conducted using the software Statistica, version 6 (Statsoft).

## Results

### TSA impairs seed germination and seedling development in *M. truncatula*

**Figure 1:**
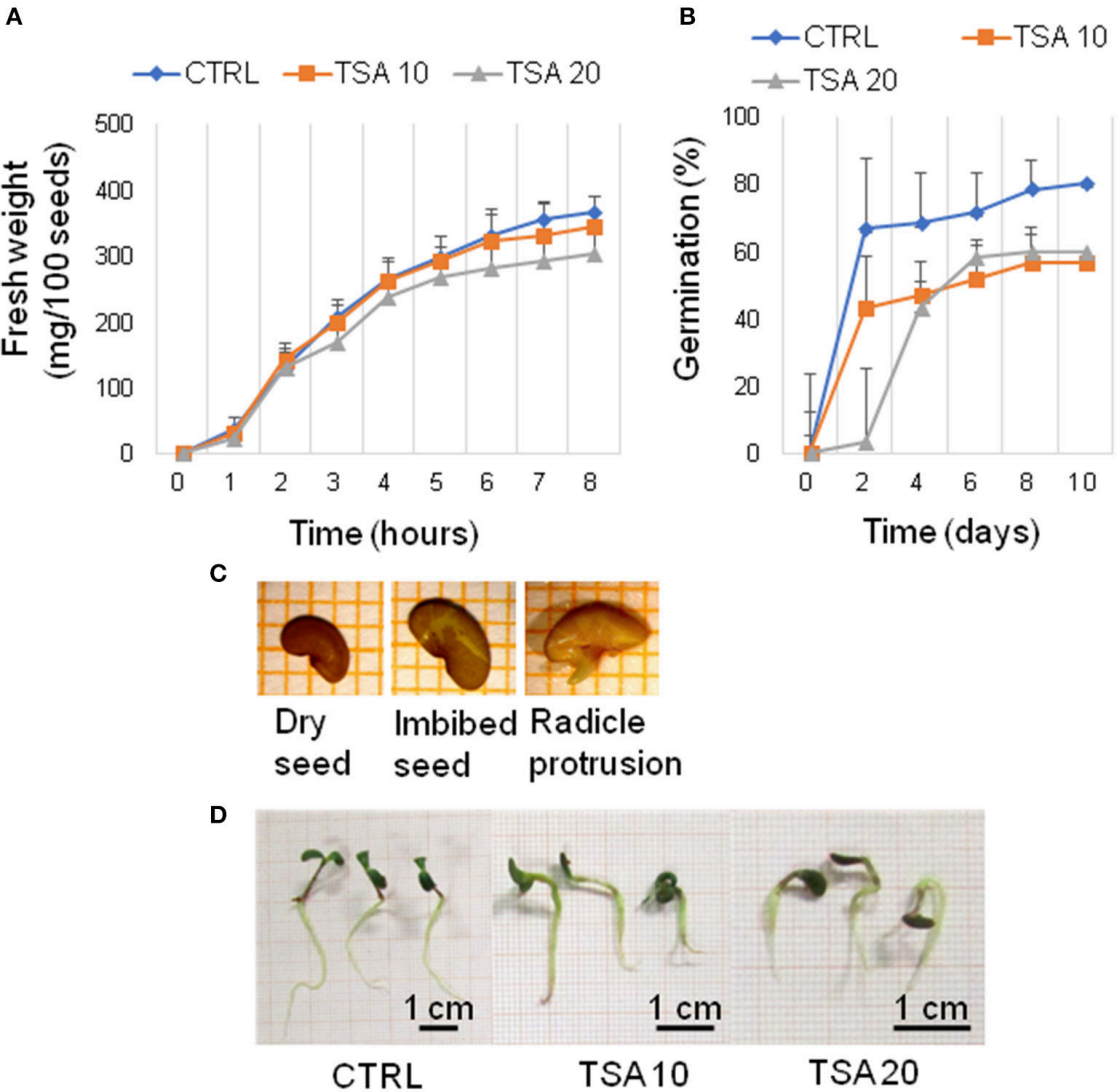
TSA delays seed germination and affects seedling development in *M. truncatula*. **(A)** Increase in fresh weight occurring during *M. truncatula* seed imbibition. **(B)** Germination percentage (%) of *M. truncatula* seeds. For all treatments, seeds with protrusion of the primary root were considered germinated. **(C)** Representative images of the phenological stages investigated. **(D)** Phenotype of 7-days old *M. truncatula* seedlings. Values are expressed as mean ± SD of three independent replications with 20 seeds for each replication. CTRL, control; TSA, trichostatin A; TSA10, 10 μM TSA; TSA20, 20 μM TSA.

A preliminary screening carried out with increasing TSA concentrations (0, 5, 10, 20, 30, and 40 μM) allowed to select the most suitable range of concentrations. For TSA doses higher than 20 μM, seed germination was inhibited. The 5 μM TSA dose did not reveal significant differences in germination, compared to CTRL (data not shown). Based on this evidence, the 10 and 20 μM TSA concentrations were selected for further investigations. During imbibition, no significant differences relative to the gain in seed fresh weight were detected in the TSA-treated samples, compared to CTRL (Figure 1A). Germination percentage of CTRL was 66.66 ± 19.29% at 48 h and no significant changes were observed until the end of the experiment. Germination was affected in TSA10 and TSA20 (Figure 1B). As for TSA10, the estimated percentage of germinated seeds reached 43.33 ± 10.27% at 48 h whereas TSA20 resulted in a similar germination percentage (43.33 ± 4.71%) only at 4-days. In TSA20, a further enhancement (up to 60.00 ± 4.08%) occurred from five to 8-days (Figure 1B). The reduced germination capacity of TSA10 and TSA20, compared to CTRL, has been expressed as peak values (Table 2). *M. truncatula* dry seeds, imbibed seed (8 h) and seeds at the radicle protrusion phase are shown in Figure 1C. When radicle protrusion is observed, seed germination has been completed and seedling growth has started. Treatments of *M. truncatula* seeds with TSA significantly delayed radicle emergence. CTRL showed radicle protrusion at 18 h following imbibition while a 24 h-delay was observed in TSA-treated seeds. In this case, radicle emergence took place at 42 h following imbibition. An abnormal phenotype was observed in TSA10 and TSA20 seedlings (Figure 1D). Biometric data (radicle length, seedling fresh weight and dry weight) are reported in Table 2. Four-days old seedlings developed from CTRL seeds showed a normal phenotype. The average radicle length was significantly reduced only in TSA20, however an overall decrease in fresh weight (27.99 and 47.40%) was observed in both TSA10 and TSA20 seedlings compared to CTRL.

**Table 2:** Results of phenotyping analyses performed on *M. truncatula* seeds/seedling exposed to TSA treatments.

	**CTRL**	**10 μM TSA**	**20 μM TSA**
Peak value	6.66 ± 1.93^a^	4.33 ± 1.03^ab^	2.23 ± 0.21^b^
Radicle length [mm]	19.33 ± 0.94^a^	18.33 ± 2.49^a^	10.00 ± 2.16^b^
Fresh weight [mg/seedling]	23.32 ± 0.66^a^	16.79 ± 0.74^b^	12.27 ± 0.60^c^
Dry weight [mg/seedling]	2.56 ± 0.10^a^	2.16 ± 0.07^ab^	1.61 ± 0.31^b^

*The peak value was calculated as the highest mean daily germination (MDG) reached at any time during the germination test. Radicle length, fresh weight and dry weight were measured in 4 days-old seedlings. CTRL, untreated control. Values are expressed as mean ± SD of three independent replications with 20 seeds for each replication. Means in a row without a common superscript letter (a,b,c) differ (P ≤ 0.05), as analyzed by one-way ANOVA*.

### Genotoxic effects of TSA and DNA repair response in *M. truncatula* seeds/seedlings

**Figure 2:**
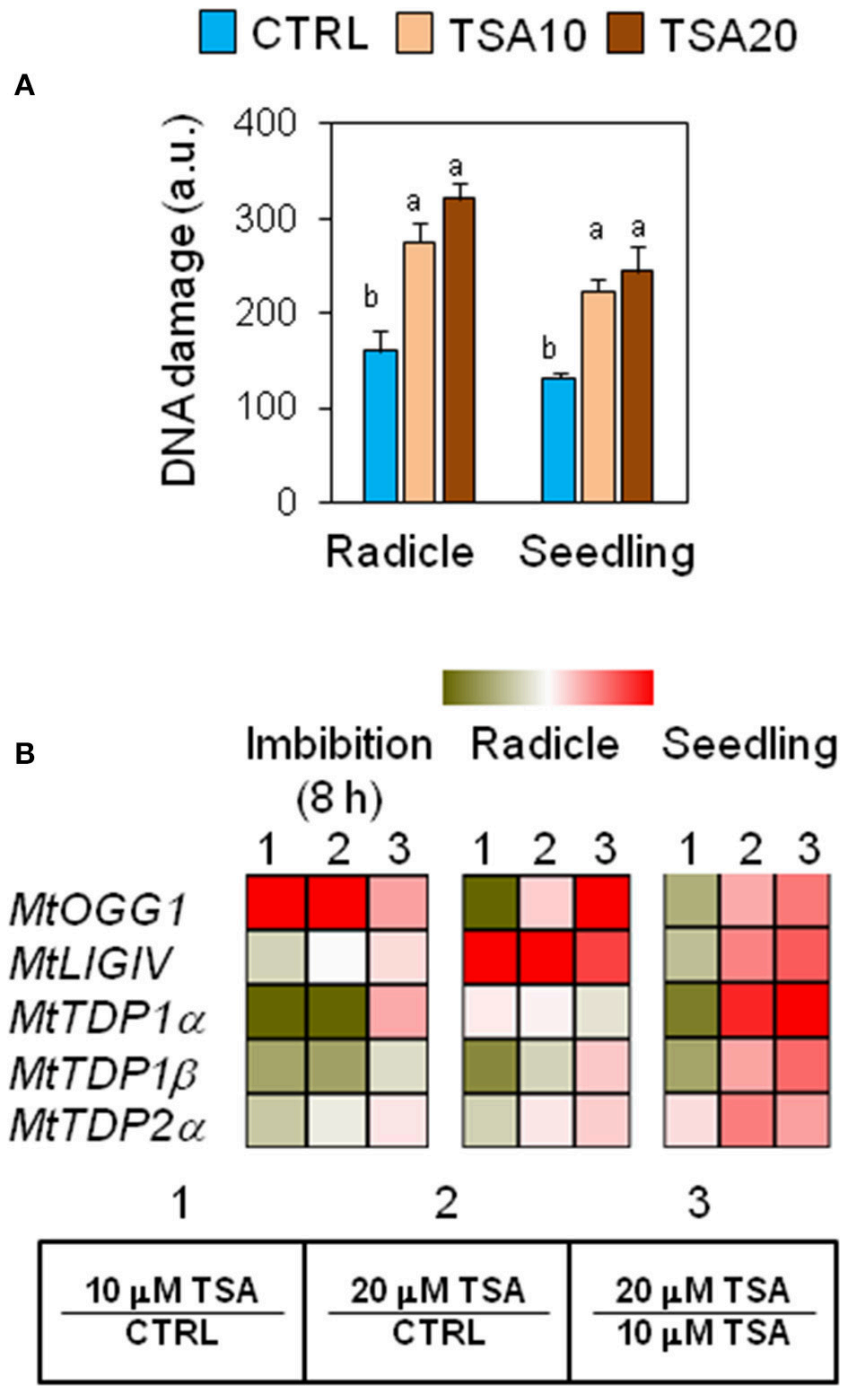
Genotoxic effects of TSA and the DNA repair response in *M. truncatula* seeds/seedlings. **(A)** Alkaline comet assay was used to measure accumulation of DNA strand breaks in seeds at the radicle protrusion phase and in 4-days old seedlings. a.u., arbitrary units. Values are expressed as mean ± SD of three independent replications with 20 seeds for each replication. Letters indicate statistically significant differences determined using One-way ANOVA (*P* < 0.05). **(B)** Heatmaps representing expression changes of DDR genes (*MtOGG1, MtLIGIV, MtTDP1*α, *MtTDP1*β, and *MtTDP2*α) at different phenological stages (imbibition 8 h; radicle; seedling) in CTRL and TSA-treated samples. Values are Log2 ratios (fold changes-FC) of transcript levels monitored by *q*RT-PCR where 1 = TSA10/CTRL, 2 = TSA20/CTRL, 3 = TSA20/TSA10. Green tones represent genes down regulated (Log2 FC ≤ 0), while red tones represent genes up-regulated (Log2 FC ≥ 0), at the specific comparison stated. Expression mean values are available in Supplemental Data (Supplemental Tables S1–S4). TSA, trichostatin A; TSA10, 10 μM TSA; TSA20, 20 μM TSA.

The genotoxic effects of TSA were assessed by performing alkaline comet assay. Total DNA strand breaks were measured in radicles isolated from CTRL and TSA-treated seeds collected at the radicle protrusion phase as well as in 4-days old seedlings. Results are shown in Figure 2A. The estimated DNA damage in CTRL radicles was 159.72 ± 21.27 a.u. (arbitrary units) while a significant increase was observed in TSA10 and TSA20 radicles (274.79 ± 21.51 a.u. and 320.60 ± 17.11 a.u., respectively). Similarly, 4-days old seedlings revealed enhanced DNA damage in response to TSA (223.83 ± 13.00 a.u. and 244.85 ± 26.86 a.u., for TSA10 and TSA20, respectively), compared to CTRL (131.82 ± 5.02 a.u.) (Figure 2A).

To verify whether TSA-induced DNA damage was able to trigger repair, the expression profiles of *MtOGG1* gene, encoding the 8-oxoguanine glycosylase/lyase enzyme, were tested. OGG1 is a key component of the BER (base excision repair) pathway involved in the removal of oxidative DNA lesions. As shown in Figure 2B, significant up-regulation in *MtOGG1* gene expression was observed at 8 h of imbibition in TSA10 (Log2 FC = 2.0) and TSA20 (Log2 FC = 2.8), compared to CTRL. In both radicles and seedlings, significant up-regulation occurred only in TSA20 (Log2 FC = 0.8) (Figure 2B). DNA ligase IV is another crucial DDR player, with specific roles in the non-homologous end joining (NHEJ) pathway responsible for DSBs repair. Significant up-regulation of *MtLIGIV* gene was observed for TSA20 at 8 h of imbibition (Log2 FC = 0.4, compared to CTRL) and in all TSA-treated radicles (Log2 FC = 3.7 and 6.4 for TSA10 and TSA20, respectively, compared to CTRL). Increased levels of the *MtLIGIV* mRNA were also observed in TSA20 seedlings (Log2 FC = 1.2, compared to CTRL) (Figure 2B).

*MtTDP1*α and *MtTDP1*β genes encoding different isoforms of the multipurpose DNA repair enzyme Tdp1 involved in the resolution of 3′-end blocking DNA lesions (including the cytotoxic topoisomerase I/DNA covalent complexes) apparently displayed minor sensitivity to the inhibitor. *MtTDP1*α gene showed up-regulation (Log2 FC = 2.1, compared to CTRL) only in TSA20 seedlings (Figure 2B). *MtTDP1*β gene did not show significant up-regulation in response to TSA, however a drop in transcript levels occurred in TSA10 imbibed seeds and radicles (Figure 2B). In the case of *MtTDP2*α gene coding for TDP2, a key player in the processing of 5′-end blocking DNA lesions (including the stabilized topoisomerase II/DNA cleavage complex), up-regulation (Log2 FC = 1.2, compared to CTRL) was observed in TSA20 seedlings (Figure 2B). The reported data reveal the genotoxic effects resulting from TSA treatments as well as DNA repair enhancement during germination.

### Exposure to TSA increases the antioxidant response in *M. truncatula* seeds/seedlings

**Figure 3:**
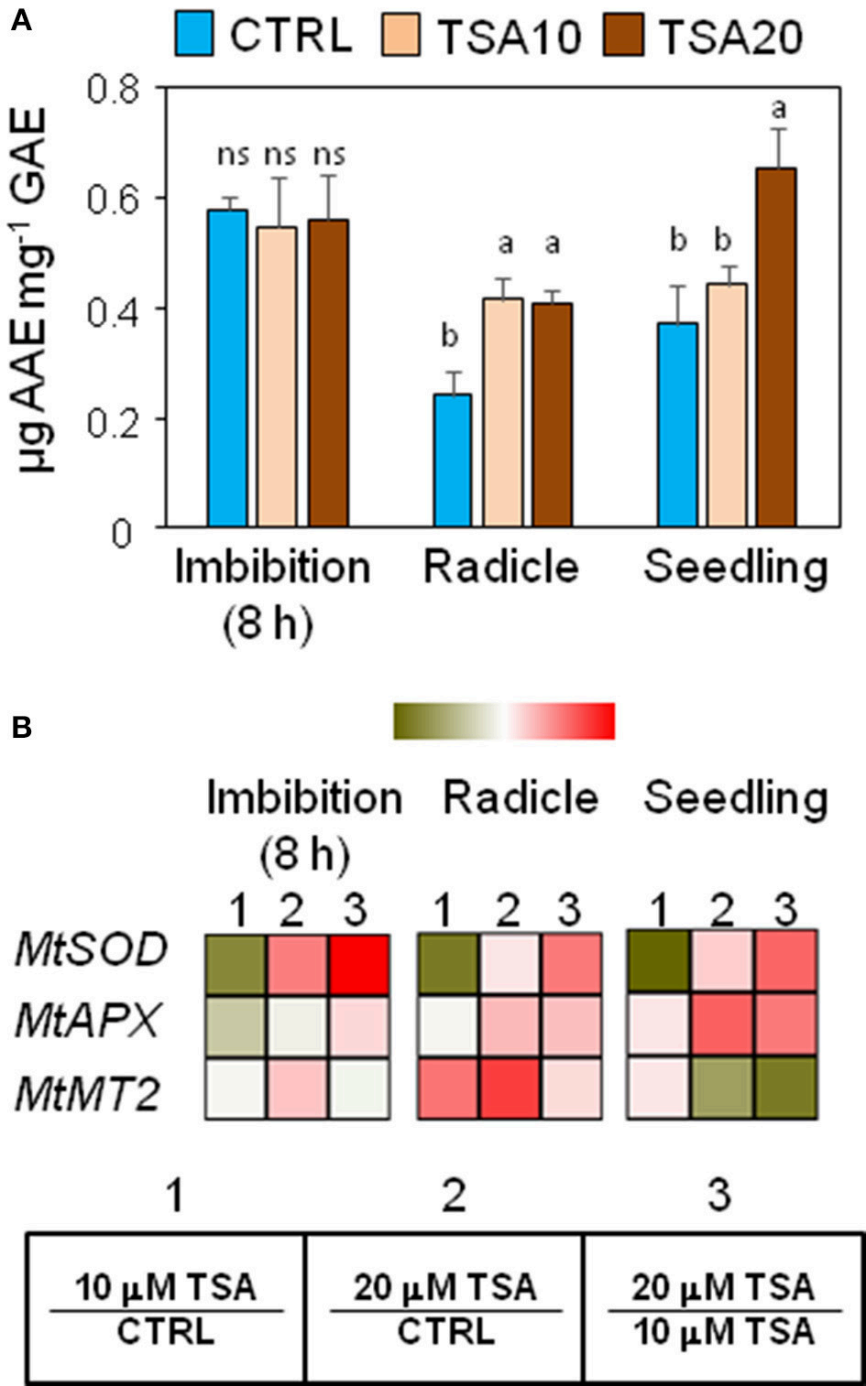
Exposure to TSA enhances the antioxidant response in *M. truncatula* seeds/seedlings. **(A)** The specific antioxidant activity of imbibed seeds (8 h), seeds at the radicle protrusion phase and 4-days old seedlings was calculated as the ratio between the antioxidant potential and the phenolic content. Values are expressed as mean ± SD of three independent replications with 20 seeds for each replication. Letters indicate statistically significant differences determined using One-way ANOVA (*P* < 0.05). **(B)** Heatmaps representing expression changes of antioxidant genes (*MtSOD, MtAPX*, and *MtMT2* at different phenological stages (imbibition 8 h; radicle; seedling) in CTRL and TSA-treated samples. Values are Log2 ratios (fold changes-FC) of transcript levels monitored by *q*RT-PCR where 1 = TSA10/CTRL, 2 = TSA20/CTRL, 3 = TSA20/TSA10. Green tones represent genes down regulated (Log2 FC ≤ 0), while red tones represent genes up-regulated (Log2 FC ≥ 0), at the specific comparison stated. Expression mean values are available in Supplemental Data (Supplemental Tables S1–S4). TSA, trichostatin A; TSA10, 10 μM TSA; TSA20, 20 μM TSA.

Changes in phenolic compounds content occurring during seed germination have been associated with fluctuations of the seed antioxidant profile. In order to verify whether phenolics might specifically contribute to the seed antioxidant response triggered by TSA, the total content in phenolic compounds as well as radical scavenging activity was assessed in *M. truncatula* seeds/seedlings using the Folin-Ciocalteu reagent and DPPH assays, respectively. Results from the DPPH test and Folin-Ciocalteau reagent method were combined to calculate the SAA expressed as μg AAE per mg GAE (Figure 3A). At 8 h of imbibition, no significant changes in the seed SAA were observed. In TSA10 and TSA20 radicles there was a significant increase in SAA, compared to CTRL whereas in seedlings a significant SAA enhancement occurred only in TSA20. The increase in SAA recorded was not associated with changes in the amount of total phenolics (data not shown). According to the reported data, the seed response to TSA did not account for phenolics-mediated antioxidant activity.

A parallel investigation was performed to assess the effects of TSA on the expression of genes with roles in the seed antioxidant response, namely *MtSOD* encoding the cytosolic isoform of superoxide dismutase, *MtAPX* coding for ascorbate peroxidase, and *MtMT2* encoding a type 2 metallothionein. In TSA10, a drop in *MtSOD* mRNA levels was observed in imbibed seeds (Log2 FC = −1.4, compared to CTRL), radicles (Log2 FC = −1.4, compared to CTRL) and seedlings (Log2 FC = −0.09, compared to CTRL) (Figure 3B). As for TSA20, a significant up-regulation was detected only in imbibed seeds (Log2 FC = 0.9, compared to CTRL) (Figure 3B). *MtAPX* gene was significantly up-regulated in TSA20 seeds (Log2 FC = 0.2, compared to CTRL) radicles and seedlings (Log2 FC = 1.1 and 1.2, compared to CTRL) (Figure 3B). *MtMT2* gene showed up-regulation in imbibed seeds (Log2 FC = 0.3 and 0.7 in TSA10 and TSA20, respectively) whereas in radicles the *MtMT2* transcript accumulated at higher levels (Log2 FC = 2.0 and 2.7 in TSA10 and TSA20, respectively). In seedlings, the level of *MtMT2* mRNA dropped in TSA20 (Log2 FC = −0.2, compared to CTRL) (Figure 3B). The emerging picture highlights the activation of antioxidant defense in response to TSA during germination.

### Influence of TSA on the expression profiles of *MtTRRAP* gene and predicted interacting partners

**Figure 4:**
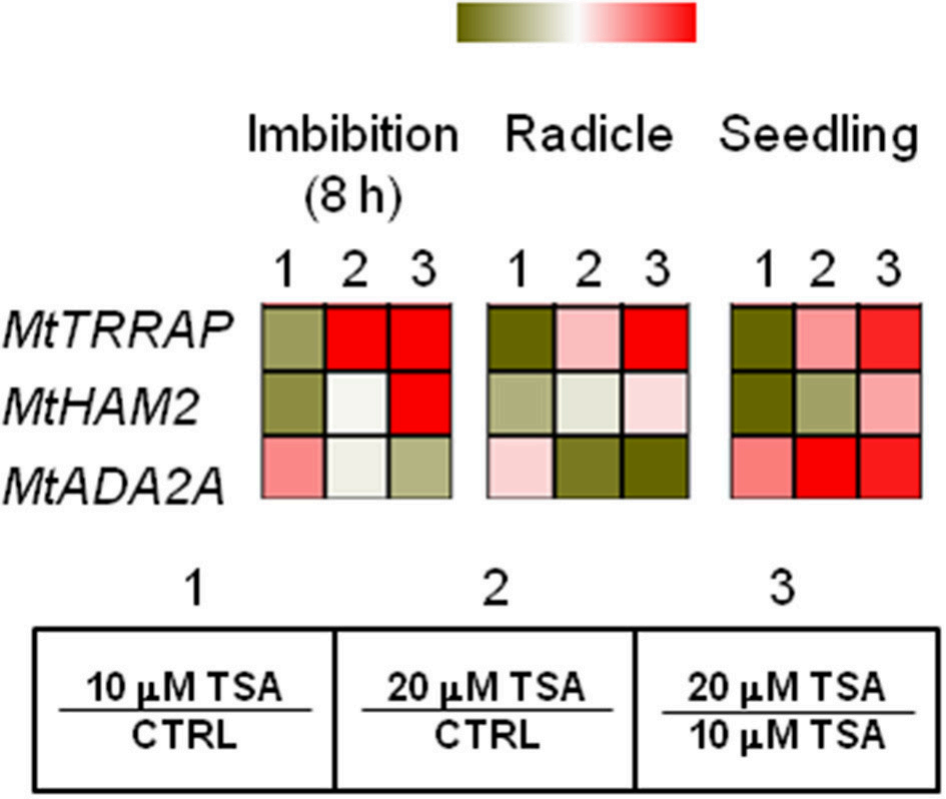
Influence of TSA on the expression of *MtTRRAP* gene and predicted interacting partners in *M. truncatula* seeds/seedlings. Heatmaps representing expression changes of chromatin remodeling genes (*MtTRRAP, MtADA2A*, and *MtHAM2* at different phenological stages (imbibition 8 h; radicle; seedling) in CTRL and TSA-treated samples. Values are Log2 ratios (fold changes-FC) of transcript levels monitored by *q*RT-PCR where 1 = TSA10/CTRL, 2 = TSA20/CTRL, 3 = TSA20/TSA10. Green tones represent genes down regulated (Log2 FC ≤ 0), while red tones represent genes up-regulated (Log2 FC ≥ 0), at the specific comparison stated. Expression mean values are available in Supplemental Data (Supplemental Tables S1–S4). TSA, trichostatin A; TSA10, 10 μM TSA; TSA20, 20 μM TSA.

As shown in Figure 4, a drop in *MtTRRAP* gene expression occurred in TSA10 at 8 h of imbibition (Log2 FC = −1.0, compared to CTRL) while transcript accumulation was observed in TSA20 (Log2 FC = 1.5, compared to CTRL). A similar profile was observed in TSA-treated radicles and seedlings. Two predicted co-expressed proteins retrieved through the *Arabidopsis* TRRAP interactome (data not shown) were also tested. These include HAM2 required for maintaining high gene expression levels at euchromatin and ADA2A, a transcriptional adaptor found in several HAT complexes. In the case of *MtHAM2* gene, a drop in the expression occurred in all the tested tissues (Log2 FC = −1.3, imbibition 8 h; Log2 FC = −0.7, radicles; Log2 FC = −1.4, seedlings) in response to the lowest TSA dose. Downregulation was also detected in TSA20 seedlings (Log2 FC = −0.5, compared to CTRL) (Figure 4). *MtADA2A* gene expression showed significant up-regulation at 8 h of imbibition in TSA10 and TSA20 (Log2 FC = 0.9 and 0.2, respectively, compared to CTRL). A drop in transcript levels occurred in TSA20 radicles (Log2 FC = −1.4, compared to CTRL) (Figure 4). A significant enhancement in *MtADA2A* mRNA was noticed in TSA10 and TSA20 seedlings (Log2 FC = 1.2 and 3.4, respectively, compared to CTRL) (Figure 4). The reported data provide insights on the response of chromatin remodeler genes with roles in DNA repair so far poorly investigated in the context of seed germination.

### TSA triggers up-regulation of proliferation marker genes and master regulators of embryo-to-seedling transition

**Figure 5:**
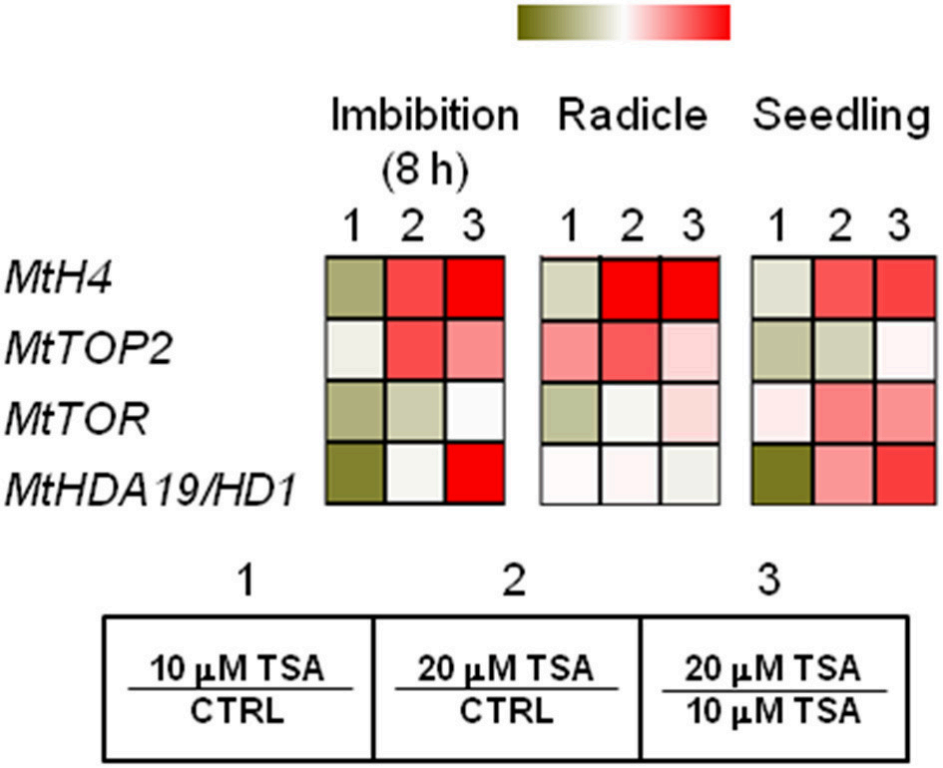
TSA modulates the expression of proliferation markers and master regulators of embryo-to-seedling transition. Heatmaps representing expression changes of proliferation marker genes (*MtH4, MtTOP2*) and master regulators of embryo-to-seedling transition (*MtTOR, MtHDA19/HD1*) at different phenological stages (imbibition 8 h; radicle; seedling) in CTRL and TSA-treated samples. Values are Log2 ratios (fold changes-FC) of transcript levels monitored by *q*RT-PCR where 1 = TSA10/CTRL, 2 = TSA20/CTRL, 3 = TSA20/TSA10. Green tones represent genes down regulated (Log2 FC ≤ 0), while red tones represent genes up-regulated (Log2 FC ≥ 0), at the specific comparison stated. Expression mean values are available in Supplemental Data (Supplemental Tables S1–S4). TSA, trichostatin A; TSA10, 10 μM TSA; TSA20, 20 μM TSA.

*MtH4* and *MtTOP2* genes were used as proliferation markers. As for *MtH4* gene, significant up-regulation was observed in TSA20 imbibed seeds (Log2 FC = 1.2, compared to CTRL), radicles (Log2 FC = 4.6, compared to CTRL), and seedlings (Log2 FC = 1.6, compared to CTRL) (Figure 5). As for *MtTOP2*, significant transcript accumulation occurred in TSA20 seeds (Log2 FC = 1.2, compared to CTRL) as well as in TSA10 and TSA20 radicles (Log2 FC = 1.6 and 2.3, respectively, compared to CTRL) (Figure 5). The TOR (TARGET OF RAPAMYCIN) protein, member of the PIKK (phosphatidylinositol kinase-related kinases) family, participates in the highly conserved signaling transduction pathways controlling embryogenesis, meristem activation, root and leaf growth. In imbibed seeds, *MtTOR* gene expression dropped (Log2 FC = −0.7 and −0.2 for TSA10 and TSA20, compared to CTRL). In TSA10 radicles a drop occurred (Log2 FC = −0.5, compared to CTRL) whereas an increase was observed TSA20 radicles (Log2 FC = 0.1, compared to CTRL) (Figure 5). As for seedlings, up-regulation was detected in both TSA10 and TSA20 (Log2 FC = 0.1 and 1.2, respectively, compared to CTRL) (Figure 5). The *MtHDA19/HD1* gene encodes a RDP3-type HDAC with a key role in the control of embryo-to-seedling transition during germination. As for imbibed seeds, the *MtHDA19/HD1* gene was significantly downregulated in TSA10 (Log2 FC = −1.5, compared to CTRL) and up-regulated in TSA20 (Log2 FC = 0.3, compared to CTRL). Up-regulation occurred also in radicles (Log2 FC = 0.2 and 0.3 for TSA10 and TSA20, compared to CTRL). Finally, downregulation of *MtHDA19/HD1* gene was detected in TSA10 seedlings (Log2 FC = −0.8, compared to CTRL) whereas up-regulation was triggered in TSA20 seedlings (Log2 FC = 1.0, compared to CTRL) (Figure 5). Taken together, the reported data reveal some interesting molecular aspects related to proliferation and embryo-to-seedling transition under TSA treatments.

### PCA and correlation analyses disclose putative links between antioxidant players, cell proliferation markers, chromatin remodeling and DNA repair

**Figure 6:**
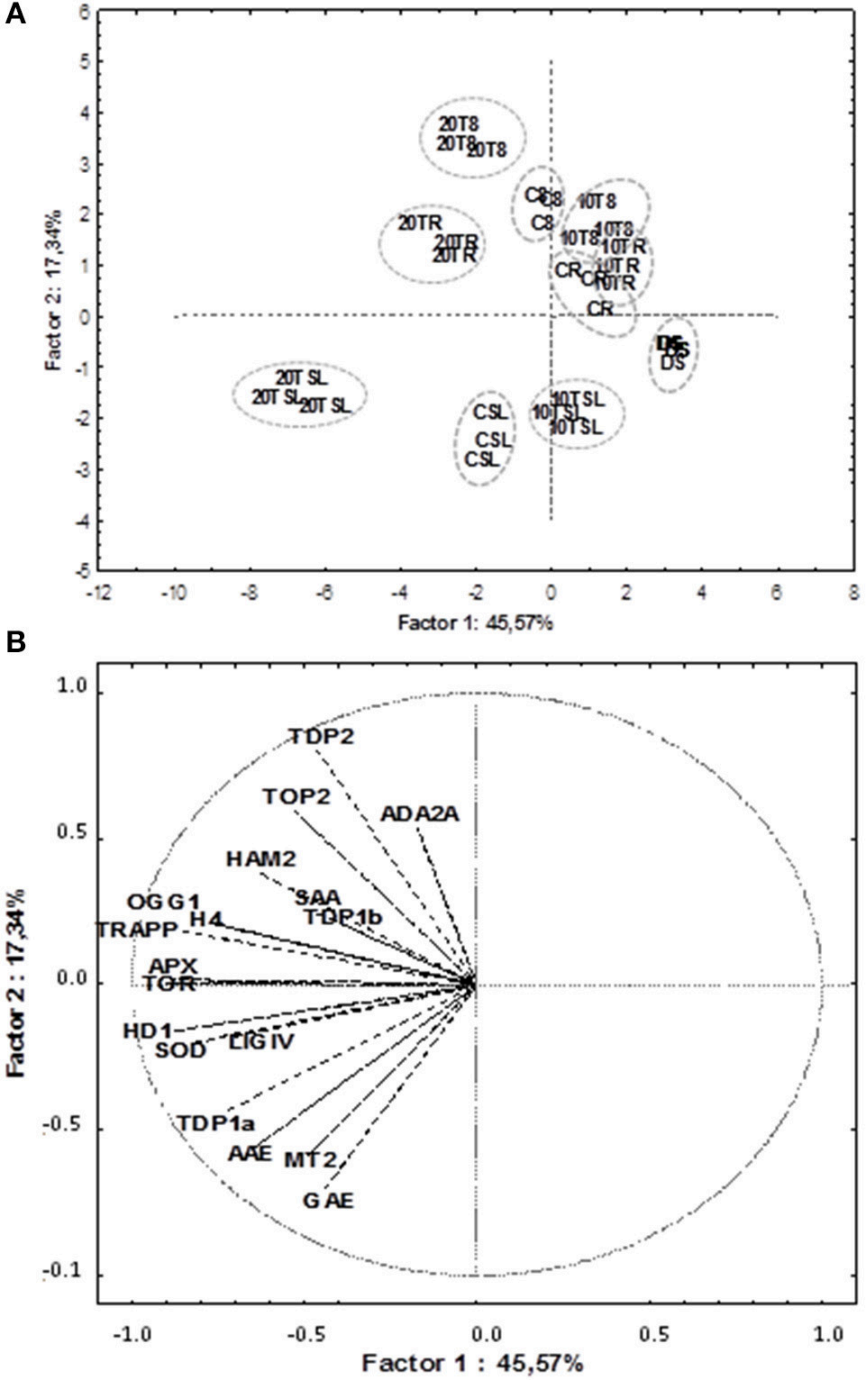
Principal component analysis of gene expression and biochemical profiles of *M. truncatula* seeds germinated in presence/absence of TSA. Data were collected on dry seeds (DS), 8 h of imbibition (8), radicle protrusion (R) and seedlings (SL) samples. **(A)** PCA scatterplot, in which the two first components explain 62.9% of the variance. **(B)** Position of the variables projected in the plane as determined by the first two principal components. Samples: DS (non-treated dry seeds); C8 (TSA10, 8 h imbibition); CR (CTRL, radicle protrusion); CSL (CTRL, seedling); 10T8 (TSA10, 8 h imbibition); 10TR (TSA10, radicle protrusion); 10TSL (TSA10, seedling); 20T8 (TSA20, 8 h imbibition); 20TR (TSA20, radicle protrusion); 20TSL (TSA20, seedling). Variables: content of total phenolic compounds (GAE); radical-scavenging activity (AAE); specific antioxidant activity (SAA); expression levels of *MtAPX, MtMT2, MtH4, MtHD19/HD1, MtTOP2, MtTOR, MtTRAPP, MtHAM2, MtADA2A, MtOGG1, MtTDP1*α, *MtTDP1*β, *MtTDP2*α, and *MtLIGIV* genes. CTRL, control; TSA, trichostatin A; TSA10, 10 μM TSA; TSA20, 20 μM TSA.

PCA was used to investigate how one sample is different from another one, which variables contribute most to this difference, and whether those variables are correlated to each other. Three main factors/components were extracted, which accounted for a 73% of the variance. Factor one accounted for 45.57% of the total variance. Variables APX (−0.89), TOR (−0.90), HDA19/HD1 (−0.88), TRAPP (−0.87) and SOD (−0.83) are strongly correlated with this component, while variables OGG1 (−0.77) and H4 (−0.78) are correlated to a lesser extent. Approximately 17.33% of the variation was assigned to factor 2, which showed correlations with TDP2 (−0.80), GAE (−0.70) and in a lesser extend MT2 (−0.60). Factor 3 accounted for only 10.9% of the variation, being correlated mainly with SAA (0.72) and TDP1β (0.65). The data were plotted according to PC1 and PC2 (Figures 6A,B), which allowed a clear separation of the majority of the samples according with phenological stage and treatment imposed. Two exceptions were noticed for the non-treated radicle samples (CR) and TSA10 at 8 h of imbibition (10T8) and TSA10 radicle (10TR) samples clustered together. The analysis of the PCA scatter plot (Figure 6A) highlights the dose dependent effect of TSA. Indeed, only the 20 μM TSA dose triggered strong effects on biochemical and gene expression profiles of treated seeds, as seen by the clear separation of the TSA20 samples from the control and 10 μM TSA. The PCA carried out using biochemical and expression data collected for the studied samples showed that correlation among variables exists. This aspect was further extended in correlation analysis (Table 3). Focusing in the most relevant cases, AAE is significantly correlated with GAE (0.83) and TDP1α (0.75), while SOD is significantly correlated with APX (0.86), TOR (0.74), HDA19/HD1 (0.83) and OGG1 (0.79). APX is correlated with TOR (0.85), HDA19/HD1 (0.88) and OGG1 (0.76). TRAPP is correlated with OGG1 (0.77) and H4 (0.83). Besides the correlation with AAE, TDP1α is also correlated with HDA19/HD1 (0.80). Overall, results suggest for new putative links between antioxidant stress responses, cell proliferation, chromatin remodeling and DNA repair.

**Table 3:** Matrix of Pearson correlation coefficients (*r*) of all biochemical and gene expression variables used in this study (*N* = 36).

**Variable**	**AAE**	**GAE**	**SAA**	**SOD**	**APX**	**MT2**	**TRAPP**	**HAM2**	**ADA2A**	**TOR**	**H4**	**HDA19/HD1**	**TOP2**	**OGG1**	**TDP1α**	**TDP1β**	**TDP2α**	**LIGIV**
AAE	1.00																	
GAE	0.83^***^	1.00																
SAA	0.44^**^	−0.07	1.00															
SOD	0.44^**^	0.46^**^	−0.03	1.00														
APX	0.43^**^	0.24	0.26	0.86^***^	1.00													
MT2	0.67^***^	0.70^***^	−0.03	0.55^***^	0.51^**^	1.00												
TRAPP	0.43^**^	0.25	0.44^**^	0.68^***^	0.66^***^	0.19	1.00											
HAM2	0.30	0.29	0.28	0.43^**^	0.41^*^	0.17	0.68^***^	1.00										
ADA2A	−0.09	−0.25	0.30	−0.02	0.27	−0.20	0.06	0.23	1.00									
TOR	0.61^***^	0.41^*^	0.40^*^	0.74^***^	0.85^***^	0.48^**^	0.66^***^	0.57^*^	0.34^*^	1.00								
H4	0.25	0.02	0.43^*^	0.65^***^	0.71^***^	0.23	0.83^***^	0.42^*^	−0.06	0.56^***^	1.00							
HD1	0.59^***^	0.45^**^	0.29	0.83^***^	0.88^***^	0.52^***^	0.66^***^	0.49^**^	0.26	0.86^***^	0.52^***^	1.00						
TOP2	0.00	−0.10	0.29	0.34^*^	0.50^**^	0.18	0.51^***^	0.64^***^	0.35^*^	0.44^**^	0.56^***^	0.34^*^	1.00					
OGG1	0.23	0.17	0.09	0.79^***^	0.76^***^	0.28	0.77^***^	0.47^**^	0.10	0.60^***^	0.78^***^	0.58^***^	0.60^***^	1.00				
TDP1α	0.75^***^	0.49^**^	0.46^**^	0.61^***^	0.65^***^	0.39^*^	0.54^***^	0.13	0.07	0.69^***^	0.41^*^	0.79^***^	−0.07	0.33	1.00			
TDP1β	0.32	0.10	0.50^**^	0.10	0.18	−0.19	0.34^*^	0.40^**^	0.10	0.43^**^	0.20	0.25	0.05	0.11	0.37^*^	1.00		
TDP2α	−0.05	−0.22	0.43^**^	0.17	0.39^*^	−0.18	0.52^***^	0.70^***^	0.53^***^	0.46^**^	0.42^**^	0.25	0.71^***^	0.47^**^	−0.07	0.45^**^	1.00	
LIGIV	0.59^***^	0.27	0.63^***^	0.50^**^	0.52^***^	0.23	0.71^***^	0.18	−0.17	0.48^**^	0.76^***^	0.51^**^	0.18	0.46^**^	0.73^***^	0.36^*^	0.08	1.00

* P ≤ 0.05;

** P ≤ 0.01;

*** *P ≤ 0.001*.

## Discussion

The multifaceted seed repair response preserves genome integrity and seed vigor. A myriad of non-enzymatic and enzymatic antioxidant components provide seeds with effective free radical scavenging activities, allowing to withstand oxidative injury during imbibition (Wojtyla et al., 2016). When the antioxidant response is properly enhanced under oxidative stress conditions, accumulation of oxidative DNA damage can be limited. The DDR network, that positively influences seed vigor (Waterworth et al., 2009, 2010, 2016; Macovei et al., 2010, 2011; Balestrazzi et al., 2011), has been so far dissected *in planta*, revealing multiple DNA damage sensing/transduction pathways that trigger lesion-specific repair (Manova and Gruszka, 2015; Hu et al., 2016; Spampinato, 2017). Another key player in this context is chromatin remodeling which significantly influences DNA repair (Donà and Mittelsten Scheid, 2015). Notwithstand the expanding knowledge, several aspects of DDR in the context of seed germination still need to be elucidated.

The present work provides for the first time the molecular profiling of the seed repair response triggered by TSA in *M. truncatula*, highlighting the effects in terms of DNA damage accumulation/ repair and antioxidant response while integrating information on chromatin remodelers. DNA lesions can be induced by HDACs inhibitors directly or indirectly, by promoting free radical accumulation (Feng et al., 2007). In animal cells, TSA can differently influence DDR, as reported in TSA-treated bladder cancer cells where the expression of DDR genes was decreased, impairing genome stability (Li et al., 2016). Up-regulation of *MtOGG1* gene in *M. truncatula* seeds/seedlings exposed to TSA suggests for the activation of the BER pathway in response to the increased DNA damage accumulation. In animal cells, the TSA-dependent acetylation of OGG1 and other BER proteins has been reported (Muftuoglu et al., 2008) whereas additional studies should be performed to unravel the TSA-mediated effects on the BER pathway in plant cells. TSA also triggered up-regulation of *MtLIGIV* gene. Distinct DNA ligases mediate the rejoining of SSBs and DSBs, an essential step in DNA repair, however DNA ligase IV is one of the major determinant of seed quality and longevity (Waterworth et al., 2010). Studies on mouse embryonic fibroblasts lacking specific NHEJ components, among which DNA ligase IV, showed increased sensitivity to TSA, thus indicating that this enzyme is required for cell survival upon exposure to the inhibitor (Yaneva et al., 2005). Most of the DDR players so far functionally characterized are highly conserved in plants and animals, however some intriguing differences have been observed in plants, compared to animals (Yoshiyama et al., 2013). As for investigations performed with HDACs inhibitors in plant cells, it should be expected that the conserved features of DDR might facilitate a better understanding of the complex molecular networks touched by these compounds or even expand the current knowledge, paving the way to interdisciplinary research (Nikitaki et al., 2017).

DDR relies on the multipurpose TDP enzymes with a specific role in the repair of topoisomerase (topo)-mediated DNA lesions as well as in the removal of a range of other 3′-end and 5′-end blocking lesions (Pommier et al., 2014, 2016). TDPs have been characterized as components of abiotic stress responses *in planta* (Macovei et al., 2010; Balestrazzi et al., 2011, 2015; Confalonieri et al., 2013; Donà et al., 2013a; Faè et al., 2014; Araujo et al., 2016b) and for their role during seed imbibition (Macovei et al., 2010; Balestrazzi et al., 2011). The observed fluctuations in the expression of *TDP* genes reflect the dose- and tissue-dependent effects of TSA and deserve further investigation, also in view of recent works in animal cells. Information concerning the effects of HDACs inhibitors on the TDP function is still scanty in animals. Duffy et al. (2017) showed that human cells overexpressing the *TDP1* gene were more sensitive to TSA whereas Meisenberg et al. (2017) suggested that TSA influences the repair of DSBs resulting from topo I/DNA cleavage complexes through a TDP1/TDP2-independent mechanism. The study by Pang et al. (2016) showed that TSA induces DSBs in human cancer cells, triggering at the same time the *RAD9*(*RADIATION SENSITIVE*) gene expression through promoter hyperacetylation. RAD9 is part of the 9-1-1 complex, early sensor of genotoxic stress, able to control G_1_/S transition. Robert et al. (2016) reported that TSA mediates acetylation of Ku70/Ku80 and polyADP-ribose polymerase-1 (PARP1), decreasing NHEJ-mediated DSBs repair. As for plants, knowledge is still limited and results hereby presented provide an interesting starting point to investigate the effects of HDACs inhibitors on different DDR pathways in the context of seed germination.

The *MtTOP2* gene was up-regulated in *M. truncatula* radicles. Zhang et al. (2016) showed that the HDAC inhibitor sodium butyrate causes the block of cell cycle at preprophase in maize root meristems, without affecting DNA integrity. Authors suggest that sodium butyrate might generate ROS acting as signaling molecules that trigger stress adaptation. Downregulation of *TOP1* and *TOP2* genes was also observed in maize root meristems. Zhang et al. (2016) hypothesized that inhibition of topoisomerases might be a critical step in stress adaptation.

HDACs inhibitors trigger ROS accumulation, leading to cell death (Robert and Rassool, 2012) and this has been reported for TSA delivered to plant cells (Jadko, 2015). The up-regulation of antioxidant genes might contribute to buffer the genotoxic effects of TSA, as demonstrated by Wang et al. (2015). Both *MtAPX* and *MtSOD* genes, required for H_2_O_2_ and superoxide radical scavenging, were previously found to be up-regulated in *M. truncatula* seeds during imbibition under physiological conditions and in response to osmotic stress (Balestrazzi et al., 2011; Macovei et al., 2011) while previous work showed that the *MtMT2* gene is also involved in the seed antioxidant response (Donà et al., 2013b). Hou et al. (2015) reported that the *SOD* promoter was hyperacetylated in maize aleurone cells during germination, as a result of gibberellin influence while treatments with ABA and TSA caused reduced acetylation and impairment of germination. In animal cells, the antioxidant activity of metallothioneins is modulated at the gene promoter level following TSA treatment (Ghoshal et al., 2002).

The observed expression profiles of *MtTRRAP* gene and its predicted interactors *MtHAM2* and *MtADA2A* might be the direct consequence of the global increase of chromatin acetylation triggered by the inhibitor or this might be part of the response to the stressful conditions caused by TSA (Chinnusamy and Zhu, 2009). No information is currently available on the TRRAP distribution within the main HAT complexes in plants. Similarly, knowledge concerning the role of TRRAP and other chromatin remodeling genes in the plant DDR is missing.

The expression profiles of the proliferation marker genes *MtH4* and *MtTOP2* observed in CTRL are in agreement with previous reports (Xie and Lam, 1994; Potokina et al., 2002). Both *MtH4* and *MtTOP2* genes were up-regulated in response to TSA, resembling animal cells where HDACs inhibitors were found to stimulate the expression of histone genes (Cuisset et al., 1998). Similarly, Chen et al. (2016) showed up-regulation of *TOP2* gene expression in human bone marrow mononuclear cells exposed to TSA. It is also known that HDACs associate *in vivo* with DNA topoisomerase II (Tsai et al., 2000) and topo II redistribution from heterochromatin was observed in TSA-treated mouse and human cell lines (Cowell et al., 2011).

Proliferation and development are strictly linked to each other and the transition from embryonic program to seedling vegetative growth is a crucial step under the control of HDA19/HD1. The latter acts sinergistically with HDA6 to activate the transcriptional switch required for vegetative growth (Tanaka et al., 2008). Fluctuations in *MtHDA19/HD1* transcript were observed in both CTRL and TSA-treated samples. Moreover, the effects of TSA on the expression of HDACs genes can vary, as demonstrated by Hemmatazad et al. (2009) who found that a class I HDAC was significantly up-regulated in animal cells treated with the inhibitor. TSA-induced up-regulation of *MtTOR* gene was evident in TSA20 seedlings. It has been previously reported that the *Arabidopsis TOR* gene is expressed throughout all the embryonic stages while in seedlings the *TOR* mRNA is accumulated in the primary meristems. The TOR-dependent phosphorylation signaling pathway has a central role in the control of germination, although several aspects of this molecular network still need to be elucidated (Deprost et al., 2007).

**Figure 7:**
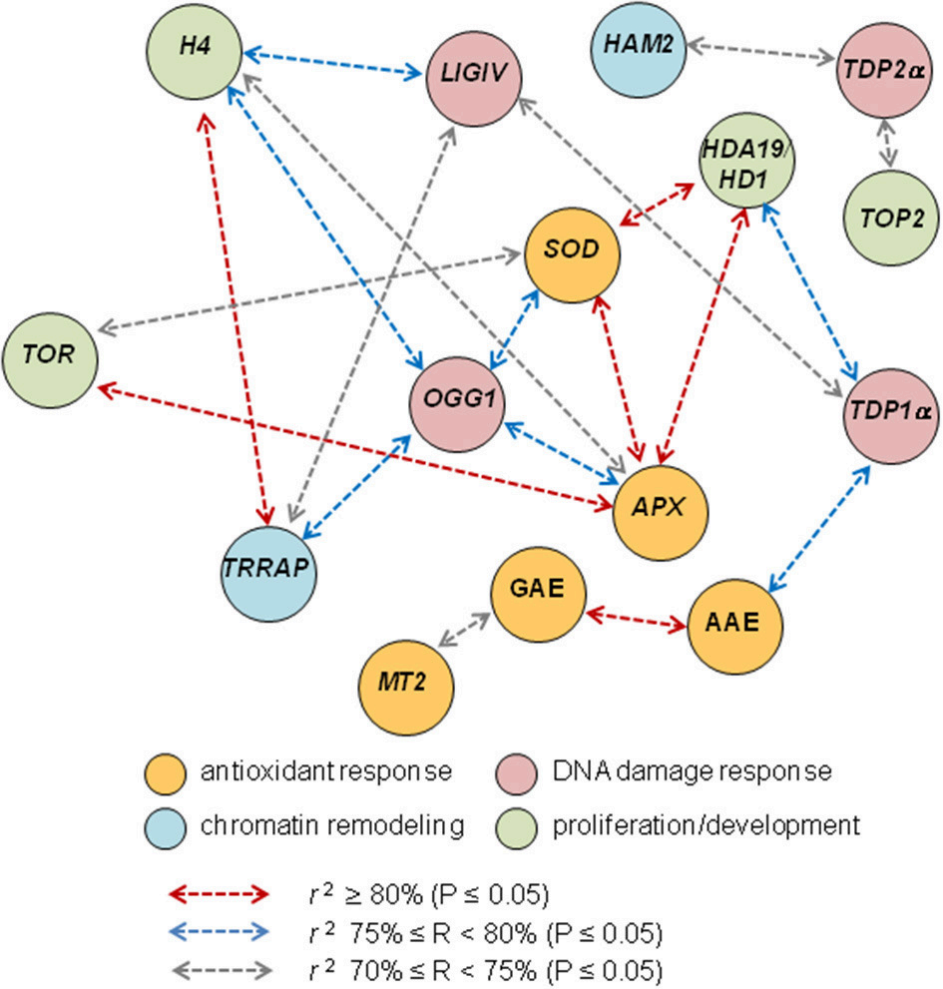
Correlation analysis discloses novel putative links between DNA repair, chromatin remodeling, antioxidant response and proliferation markers. Schematic representation of the most relevant cases of correlation of biochemical and gene expression variables based on the Pearson correlation coefficients *r* listed in Table 3.

Finally, correlation analysis predicted a robust link between the *MtTRRAP* and *MtOGG1* functions (Figure 7) which appear to bridge DNA repair and chromatin remodeling in the context of seed germination. This is a novel clue to the elucidation of the seed repair response, evidenced by the TSA treatments, that might deserve further investigation. Notably, *MtOGG1* correlates also with the *MtAPX* and *MtSOD* (Figure 7), strengthening the link between DNA repair and antioxidant response, a crucial aspect of seed vigor.

## Conclusions

The present work brings the attention to the entangled correlations that link DNA repair, antioxidant response, and chromatin remodeling in the specific context of seed germination, using TSA as stress agent to disclose sensitive targets. The emerging picture looks extremely complicated since TSA, acts in different ways at the cellular level. The inhibitor blocks cell cycle, induces ROS accumulation and DNA damage, and all these events touch directly or indirectly molecular processes that contribute to seed vigor. Results hereby shown are snapshots of the whole picture which is evidently much wider. To better understand this scenario, it is essential to integrate the discussion on plant data with the current knowledge available in animal systems where the role(s) of HDACs inhibitors in DDR, chromatin remodeling and ROS metabolism, is intensively investigated. Intriguing issues are raised by the study of the seed response to TSA in *M. truncatula*, all of them deserving future in-depth investigation. Does DDR in seeds account for repair pathways that are preferentially activated in response to chromatin perturbation? Is there any cross-talk involving the seed repair machinery and the master regulators of cell proliferation/seedling development? Once addressed, these questions will open concrete perspectives in basic and applied seed biology.

## Author contributions

AB and SA conceived the work and wrote the manuscript; AP, AM, and PL contributed the experimental data; AP performed bioinformatic analyses; AB, SA, PL, AP, and AM contributed to the discussion of data. All authors read and agreed with the final version of the manuscript.

### Conflict of interest statement

The authors declare that the research was conducted in the absence of any commercial or financial relationships that could be construed as a potential conflict of interest.
