# Supplementary material for: The Seed Repair Response during Germination: Disclosing Correlations between DNA Repair, Antioxidant Response, and Chromatin Remodeling in Medicago truncatula
Source: Front Plant Sci. 2017 Nov 14;8:1972. doi: 10.3389/fpls.2017.01972 (PMC5694548; doi:10.3389/fpls.2017.01972)
Supplement: Supplementary file 1 [file DataSheet1.pdf]

Supplemental Data

Using trichostatin A to disclose the seed repair response during germination in *Medicago truncatula*

Andrea Pagano, Susana de Sousa Araújo, Anca Macovei, Paola Leonetti, Alma Balestrazzi

Supplemental Table S1. Relative gene expression values measured in dry seeds of *M. truncatula*. Data are means±SD from three biological replicates.

Dry seed	CTRL
**SOD**	1.633 ± 0.152
**APX**	0.572 ± 0.182
**MT2**	0.037 ± 0.013
**TRAPP**	0.201 ± 0.015
**HAM2**	0.253 ± 0.063
**ADA2A**	0.047 ± 0.007
**TOR**	0.053 ± 0.013
**H4**	0.535 ± 0.106
**HD1**	0.054 ± 0.038
**TOP2**	0.001 ± 0.000
**OGG1**	0.203 ± 0.217
**TDP1α**	0.050 ± 0.001
**TDP1β**	0.110 ± 0.045
**TDP2 α**	0.021 ± 0.005
**LIGIV**	0.651 ± 0.097

Supplemental Table S2. Relative gene expression values measured in *M. truncatula* seeds collected at 8 h of imbibition with 0 (CTRL), 10 µM TSA and 20 µM TSA. Data are means±SD from three biological replicates. Means in a row without a common superscript letter (a,b,c) differ ($P \leq 0.05$), as analyzed by one-way ANOVA.

8h imbibition	CTRL	10 µM TSA	20 µM TSA
**SOD**	2.574 ± 0.285^{b}	0.966 ± 0.255^{c}	5.133 ± 0.277^{a}
**APX**	8.527 ± 2.105	6.563 ± 0.789	10.303 ± 2.555
**MT2**	0.134 ± 0.037	0.174 ± 0.093	0.222 ± 0.027
**TRAPP**	0.403 ± 0.065^{b}	0.191 ± 0.032^{b}	1.207 ± 0.235^{a}
**HAM2**	3.376 ± 0.401^{a}	1.343 ± 0.624^{b}	4.334 ± 0.888^{a}
**ADA2A**	0.863 ± 0.167^{b}	1.675 ± 0.484^{a}	1.030 ± 0.030^{a}
**TOR**	1.483 ± 0.186^{a}	0.881 ± 0.275^{b}	1.223 ± 0.184^{ab}
**H4**	0.849 ± 0.117^{b}	0.473 ± 0.041^{b}	1.984 ± 0.470^{a}
**HD1**	0.599 ± 0.071^{b}	0.210 ± 0.031^{c}	0.774 ± 0.093^{a}
**TOP2**	0.062 ± 0.007	0.074 ± 0.053	0.142 ± 0.040
**OGG1**	0.189 ± 0.200^{c}	0.761 ± 0.269^{b}	1.384 ± 0.153^{a}
**TDP1α**	0.075 ± 0.003^{a}	0.004 ± 0.000^{b}	0.007 ± 0.003^{b}
**TDP1β**	0.291 ± 0.025^{a}	0.149 ± 0.055^{b}	0.146 ± 0.006^{b}
**TDP2 α**	0.961 ± 0.179	0.738 ± 0.047	1.122 ± 0.185
**LIGIV**	0.604 ± 0.088^{b}	0.532 ± 0.032^{b}	0.827 ± 0.027^{a}

Supplemental Table S3. Relative gene expression values measured at radicle protrusion in *M. truncatula* seeds treated with 0 (CTRL), 10 μM TSA and 20 μM TSA. Data are means±SD from three biological replicates. Means in a row without a common superscript letter (a,b,c) differ ($P \leq 0.05$), as analyzed by one-way ANOVA.

Radicle	CTRL	10 μM TSA	20 μM TSA
**SOD**	8.287 ± 1.552^{b}	3.103 ± 0.262^{c}	12.044 ± 1.622^{a}
**APX**	11.014 ± 2.331^{b}	11.788 ± 1.716^{b}	24.033 ± 1.370^{a}
**MT2**	0.619 0.018^{c}	2.546 0.292^{b}	4.078 ± 0.347^{a}
**TRAPP**	0.359 ± 0.119^{b}	0.038 ± 0.017^{c}	0.750 ± 0.101^{a}
**HAM2**	2.141± 0.112^{a}	1.293 ± 0.168^{b}	2.029 ± 0.131^{a}
**ADA2A**	1.034 ± 0.045^{a}	1.747 ± 0.713^{ab}	0.380 ± 0.290^{a}
**TOR**	1.316 ± 0.178	0.904 ± 0.162	1.438 ± 0.282
**H4**	0.108 ± 0.052^{b}	0.090 ± 0.059^{b}	2.766± 0.729^{a}
**HD1**	0.809 ± 0.061	0.977 ± 0.307	1.023 ± 0.153
**TOP2**	0.025 ± 0.013^{c}	0.077 ± 0.002^{b}	0.128 ± 0.011^{a}
**OGG1**	1.044 ± 0.121^{b}	0.111 ± 0.022^{c}	1.868 ± 0.261^{a}
**TDP1 α**	0.052 ± 0.003	0.072 ± 0.027	0.067 ± 0.015
**TDP1β**	0.122 ± 0.002	0.052 ± 0.007	0.100 ± 0.055
**TDP2 α**	0.478 ± 0.170	0.377 ± 0.037	0.683 ± 0.209
**LIGIV**	0.011 ± 0.001^{b}	0.146 ± 0.135^{b}	0.956 ± 0.119^{a}

Supplemental Table S4. Relative gene expression values measured in seedling from *M. truncatula* seeds treated with 0 (CTRL), 10 µM TSA and 20 µM TSA. Data are means±SD from three biological replicates. Means in a row without a common superscript letter (a,b,c) differ ($P \leq 0.05$), as analyzed by one-way ANOVA.

Seed	CTRL	10 µM TSA	20 µM TSA
**SOD**	9.297 ± 0.763[b]	4.576 0.143[c]	12.891 ± 2.356[a]
**APX**	9.477 ± 0.835[b]	11.292 ± 0.991[b]	27.733 ± 3.268[a]
**MT2**	6.694 ± 0.582[a]	7.876 ± 0.223[a]	4.366 ± 1.182[b]
**TRAPP**	0.576 ± 0.048[b]	0.265 ± 0.022[c]	1.176 ± 0.159[a]
**HAM2**	3.423 ± 0.472[a]	1.248 ± 0.406[b]	2.271 ± 0.373[b]
**ADA2A**	0.090 ± 0.029[b]	0.218 ± 0.041[b]	1.015 ± 0.389[a]
**TOR**	1.181 ± 0.067[b]	1.317 ± 0.384[b]	2.764 ± 0.485[a]
**H4**	0.720 ± 0.097[b]	0.640 ± 0.152[b]	2.269 ± 0.602[a]
**HD1**	1.079 ± 0.312	0.590 ± 0.111	2.177 ± 0.060
**TOP2**	0.058 ± 0.009	0.045 ± 0.016	0.048 ± 0.016
**OGG1**	0.805 ± 0.136[b]	0.564 ± 0.177[b]	1.414 ± 0.197[a]
**TDP1α**	0.197 ± 0.017[b]	0.110 ± 0.003[c]	0.861 ± 0.025[a]
**TDP1β**	0.122 ± 0.024[b]	0.081 ± 0.016[b]	0.224 ± 0.069[a]
**TDP2α**	0.187 ± 0.084	0.233 ± 0.161	0.444 ± 0.102
**LIGIV**	0.705 ± 0.041[b]	0.532 ± 0.156[b]	1.621 ±0.138[a]

Supplemental Table S5. AAE (ascorbic acid equivalents), GAE (gallic acid equivalents) and SAA (specific antioxidant activity) values measured in seeds collected at 8 h of imbibition treated with 0 (CTRL), 10 µM TSA and 20 µM TSA. Data are means±SD from three biological replicates. Means in a row without a common superscript letter (a,b,c) differ (P ≤ 0.05), as analyzed by one-way ANOVA.

8h imbibition	CTRL	10 µM TSA	20 µM TSA
**AAE**	4.941 ± 0.536	5.219 ± 0.556	4.513 ± 0.263
**GAE**	8.531 ± 0.619	9.827 ± 2.835	8.113 ± 0.820
**SAA**	0.578 ± 0.024	0.548 ± 0.089	0.562 ± 0.082

Supplemental Table S6. AAE (ascorbic acid equivalents), GAE (gallic acid equivalents) and SAA (specific antioxidant activity) values measured in seeds collected at the radicle protrusion phase treated with 0 (CTRL), 10 µM TSA and 20 µM TSA. Data are means±SD from three biological replicates. Means in a row without a common superscript letter (a,b,c) differ (P ≤ 0.05), as analyzed by one-way ANOVA

Radicle	CTRL	10 µM TSA	20 µM TSA
**AAE**	2.587 ± 0.038^{b}	2.536± 0.065^{b}	2.741 ± 0.048^{a}
**GAE**	10.953 ± 1.781^{a}	6.122 ± 0.644^{b}	6.750 ± 0.263^{b}
**SAA**	0.241 ± 0.042^{b}	0.417 ± 0.038^{a}	0.410 ± 0.021^{a}

Supplemental Table S7. AAE (ascorbic acid equivalents), GAE (gallic acid equivalents) and SAA (specific antioxidant activity)values measured in seedlings from in seeds treated with 0 (CTRL), 10 µM TSA and 20 µM TSA. Data are means ± SD from three biological replicates. Means in a row without a common superscript letter (a,b,c) differ (P≤0.05), as analyzed by one-way ANOVA

Seedling	CTRL	10 µM TSA	20 µM TSA
**AAE**	9.408 ± 0.376^{b}	8.782 ± 0.175^{b}	11.652 ± 0.568^{a}
**GAE**	25.772 ± 3.574^{a}	17.104 ± 3.596^{b}	17.910 ± 2.041^{a}
**SAA**	0.372 ± 0.070^{b}	0.443 0.033^{b}	0.656 ± 0.071^{a}
